# Tricuspid Valve Geometrical Changes in Patients with Functional Tricuspid Regurgitation: Insights from a CT Scan Analysis Focusing on Commissures

**DOI:** 10.3390/jcm12051712

**Published:** 2023-02-21

**Authors:** Valeria Cammalleri, Edoardo Nobile, Domenico De Stefano, Myriam Carpenito, Simona Mega, Maria Caterina Bono, Aurelio De Filippis, Annunziata Nusca, Carlo Cosimo Quattrocchi, Francesco Grigioni, Gian Paolo Ussia

**Affiliations:** 1 Unit of Cardiovascular Science, Fondazione Policlinico Universitario Campus Bio-Medico, Via Alvaro del Portillo, 200, 00128 Roma, Italy; 2 Unit of Diagnostic Imaging and Interventional Radiology, Fondazione Policlinico Universitario Campus Bio-Medico, Via Alvaro del Portillo, 200, 00128 Roma, Italy

**Keywords:** tricuspid valve, transcatheter tricuspid valve intervention, tricuspid valve commissures, computed tomography, multimodality imaging, atrial functional tricuspid regurgitation

## Abstract

Background: Cardiac computed tomography (CT) provides important insights into the geometrical configuration of the tricuspid valve (TV). The purpose of the present study was to assess the geometrical changes of TV in patients with functional tricuspid regurgitation (TR) using novel CT scan parameters and to correlate these findings with echocardiography. Methods: This single-center study enrolled 86 patients undergoing cardiac CT and divided them into two groups according to the presence or not of severe TR (43 patients with TR ≥ 3+ and 43 controls). The measurements collected were as follows: TV annulus area and perimeter, septal-lateral and antero-posterior annulus diameters, eccentricity, distance between commissures, segment between the geometrical centroid and commissures, and the angles of commissures. Results: We found a significant correlation between all annulus measurements and the grade of TR, except in regard to angles. TR ≥ 3+ patients had significantly larger TV annulus area and perimeter, larger septal-lateral, and antero-posterior annulus dimensions, as well as larger commissural distance and centroid-commissural distance. In patients with TR ≥ 3+ and controls, the eccentricity index predicted a circular shape and an oval shape of the annulus, respectively. Conclusions: These novel CT variables focusing on commissures increase the anatomical understanding of the TV apparatus and the TV geometrical changes in patients with severe functional TR.

## 1. Introduction

The recent increase in interest in the tricuspid valve (TV) is justified by the recognition that tricuspid regurgitation (TR) is common and adversely affects prognosis by promoting the deleterious cycle of valvular-driven heart failure [1,2,3,4]. Tricuspid valve surgery is generally underutilized in clinical practice and often proposed too late because of an unacceptably high perioperative mortality [3,5,6]. Accordingly, the need for a transcatheter-based treatment approach has become substantial, and several devices have been developed, miming mitral valve transcatheter intervention: leaflet edge-to-edge repair, direct annuloplasty, and the implant of a prosthesis in a heterotopic or orthotopic position [7]. Therefore, illustrating and describing TV anatomy through noninvasive imaging techniques is the first step in the diagnostic algorithm of patients who are candidates for transcatheter tricuspid valve intervention (TTVI). Currently, a multimodality approach, including echocardiography and computed tomography (CT), is strictly recommended for illustrating the TV anatomy, guiding the decision-making process, and supporting the procedures and the development of novel transcatheter devices [6,7,8,9,10]. Nevertheless, due to the non-planar configuration of the TV apparatus and its position in the chest, detailed morphological illustration can be challenging using echocardiography. On the contrary, cardiac CT, enabling the acquisition of high spatial resolution three-dimensional data, provides essential insights into the geometrical configuration of the TV. Each TTVI requires specific imaging protocols to evaluate the anatomical feasibility and predict outcomes after the procedure. Likewise, different CT parameters have been tested so far in order to support the screening process and the development of novel transcatheter therapies [11,12,13,14,15].

The present study aimed to evaluate the geometrical changes of the TV apparatus in patients with significant functional TR vs. controls, using novel CT scan parameters, and to correlate these findings with the TR grade assessed with echocardiography.

## 2. Materials and Methods

### 2.1. Patient Population

A total of 86 patients undergoing cardiac CT scans were enrolled in this single-center study. The entire patient population was divided into two groups according to the presence or not of severe TR. Specifically, 43 patients with symptomatic severe TR, referred to our institute for TTVI consideration, were prospectively enrolled as part of the previously published TRIMA (Tricuspid Regurgitation IMAging) study (TR ≥ 3+ group) [14]. They were retrospectively compared with 43 patients who underwent cardiac CT scanning for triple rule-out for acute coronary artery disease, aortic dissection, or pulmonary embolism when clinically indicated (control group) [16,17,18]. Exclusion criteria for both groups included the presence of active endocarditis, congenital heart disease, previous tricuspid valve surgery, and the need for urgent open cardiac surgery. For the control group, further exclusion criteria were the following: any valve heart disease of moderate or greater degree (including TR); previous any cardiac surgery or previous myocardial infarction; heart failure; right ventricle dysfunction; pulmonary hypertension; and insufficient CT image quality or contrast media in the right heart chambers to characterize the TV annulus and commissures. TR severity was quantified using the five-grade classification according to echocardiographic qualitative, semiquantitative, and quantitative methods [19,20]. CT scans were stored in institutional servers and subsequently analyzed. This study complies with the Declaration of Helsinki and was approved by the local ethics committee. All patients provided written informed consent.

### 2.2. CT Scan Acquisition

A 128-slice multidetector CT scanner (Siemens Somatom Definition AS+, Siemens Erlangen, Germany) with a collimation of 128 × 0.6 mm was used for performing CT examinations. ECG-assisted data acquisition was carried out with a retrospective ECG-gating without dose modulation, covering the entire cardiac cycle (R-R) at the same dosage. Based on the patient body mass index (BMI) and glomerular filtration rate (GFR), the tube voltage varied for each examination from 100 to 120 kV. Ionic contrast agent (Omnipaque 350 mg I/mL, GE Healthcare, USA), adjusted for BMI and GFR, was infused into the antecubital vein according to two different protocols [14,17]. In the TR ≥ 3+ population, a biphasic contrast protocol was infused—60–80 mL mixture of 80%/20% contrast/saline, with a flow rate of 4.0–5.0 mL/s, followed by a 50 mL of saline—whereas patients for the triple rule-out group (control group) received a triphasic contrast protocol—70–80 mL of contrast with a flow rate of 5.0–7.0 mL/s followed by a 50 mL mixture of 50%/50% contrast/saline with a flow rate of 4.0 mL/s and by a 30 mL of saline. In the TR ≥ 3+ group, the onset of scanning was synchronized with the arrival of the contrast agent in the main pulmonary trunk, using automated peak enhancement detection with a threshold of 120 Hounsfield units (HU). In this population, the scan target window included the entire heart, from the superior vena cava to the suprahepatic inferior vena cava level. In the control group, the onset of scanning was synchronized with the arrival of the contrast media in the ascending aorta, using automated peak enhancement detection with a threshold of 150 HU; the scan target volume included the entire thorax, from the apex to the diaphragm, with a caudo-cranial acquisition. Thus, CT datasets were reconstructed as an axial thin-sliced image (0.6–0.75 mm, with soft-tissue convolution kernel and iterative reconstruction algorithm, depending on the level of image noise) at each 5% of the R-R interval, covering the entire cardiac cycle with a multi-phase set for coronaries evaluation; for the aorta and pulmonary arteries, a thicker axial reconstruction was realized instead (1 mm with soft-tissue window and convolution kernel and iterative reconstruction algorithm, depending on the level of image noise). ECG editing was used in case of uncorrected sync.

### 2.3. CT Data Analysis

**Figure 1:**
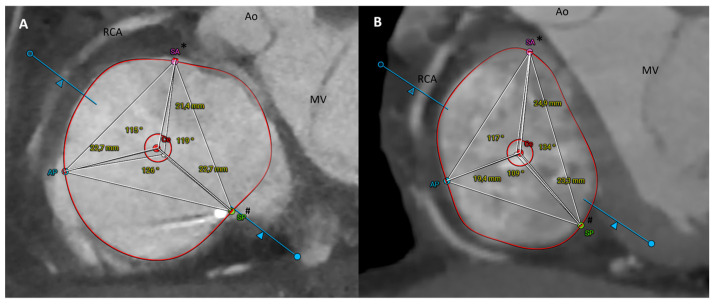
CT scan analysis using 3mensio Structural Heart 10.3 (Pie Medical Imaging, Maastricht, The Netherlands) in a patient with severe tricuspid regurgitation (**A**) and control (**B**). The different geometrical shape of the tricuspid annulus is appreciable: quite circular in (**A**) and more oval in (**B**). AP: antero-posterior commissure; Ce: centroid; SA: septal-anterior commissure SP: septal-posterior commissure. *SA corresponds to AS antero-septal commissure in the main text and tables; ^#^SP corresponds to PS postero-septal commissure in the main text and tables.

An offline analysis was performed using the tricuspid workflow of the external workstation 3mensio Structural Heart 10.3 (Pie Medical Imaging, Maastricht, The Netherlands). Mid-diastolic data (60–80% of cardiac cycle) were analyzed to reconstruct the TV geometry, using the previously published analysis protocol [14]. Briefly, the following measurements were collected: TV three-dimensional annulus area and perimeter, septal-lateral and antero-posterior annulus diameters, eccentricity index, distance between commissures, segment between the geometrical centroid (Ce) and commissure, and angles between the Ce-segments. The protocol analysis is illustrated in Figure 1 and schematized in Table 1.

### 2.4. Statistical Analysis

Categorical variables are presented as frequencies and percentages; continuous variables are expressed as mean ± standard deviation. Differences between the two groups were compared using the Χ^2^ test for categorical variables and an unpaired Student’s *t*-test for continuous variables. Pearson’s correlation explored the relationship between CT measurements and TR grade. Kruskal–Wallis analysis was used to assess the CT variables distribution across the expanded TR grades by echocardiography. Linear multiple regression was used to test commissural CT variables associated with the annulus area. To avoid multi-collinearity, correlation between these measurements and annulus area was tested separately. Results are reported as point estimates and 95% confidence intervals (CI). Differences were considered significant at *p* < 0.05. Statistical analyses were performed using IBM SPSS version 26 (IBM, Armonk, NY, USA).

## 3. Results

Among 86 patients (mean age 74.86 ± 11.49 years, 42% male), 26 (30%) had severe TR (3+), 11 (13%) had massive TR (4+), 6 (7%) had torrential TR (5+), and the other 43 patients (50%), who were part of the control group, had trace or mild TR (1+). No patients with moderate TR (2+) were enrolled. The mechanism of TR ≥ 3+ was functional in all cases (atrial 91%, ventricular 9%). Although the 41% of TR ≥ 3+ patients had a cardiac implantable electronic device (CIED), the mechanism of TR was considered to be secondary CIED-induced as a consequence of the TV remodeling, following the right ventricle dilatation and dysfunction due to pacing/heart failure [21]. Therefore, they were merely classified as functional TR.

A three-leaflet configuration of the TV was observed in all patients; 36 (41.8%) presented a multi-scalloped anterior or posterior leaflet with the three main commissures always identifiable. The clinical characteristics of the patients are presented in Table 2.

Patient groups were comparable in clinical characteristics, left ventricle ejection fraction, and body mass index. Patients with TR ≥ 3+ were significantly older, female, and prevalently affected by atrial fibrillation and rhythm disorders, requiring the implantation of a cardiac implantable electronic device.

Adequate CT images for the evaluation of TV geometry were available in all patients. The results of the CT data analysis and differences between the two groups are reported in Table 3.

Within the TR ≥ 3+ population, no significant differences in the TV geometry were observed between patients with secondary CIED-induced TR and pure functional TR (Supplementary Table S1). Conversely, our study population counted geometrical differences in TV anatomy between patients with and without TR ≥ 3+. Compared with controls, patients with at least severe TR had a significantly larger TV annulus area (16.84 ± 32.55 vs. 12.09 ± 2.37, *p* < 0.001) and perimeter (147.77 ± 17.15 vs. 129.47 ± 13.24, *p* < 0.001), as well as a larger septal-lateral (46.80 ± 6.44 vs. 36.80 ± 5.21, *p* < 0.001) and antero-posterior (45.67 ± 5.05 vs. 39.39 ± 4.97, *p* < 0.001) annulus dimension. It is of note that in patients with TR ≥ 3+, the septal-lateral annulus diameter was slightly larger than the antero-posterior one, with an eccentricity index of 0.99 ± 0.12, suggesting an almost perfectly circular shape of the TV annulus. Conversely, in the control group, the septal-lateral dimension was the smallest, with an eccentricity index of 1.11 ± 0.31, indicating an oval shape of the tricuspid valve annulus in patients without significant TR (Figure 1). Additionally, compared to controls, all commissural distances and centroid-commissural segments were larger in patients with significant TR. Remarkably, the greatest difference was in PS-AP (difference 5.49) and AP-AS distance (difference 7.03). There was no statistically significant difference in Ce-commissural angles.

**Figure 2:**
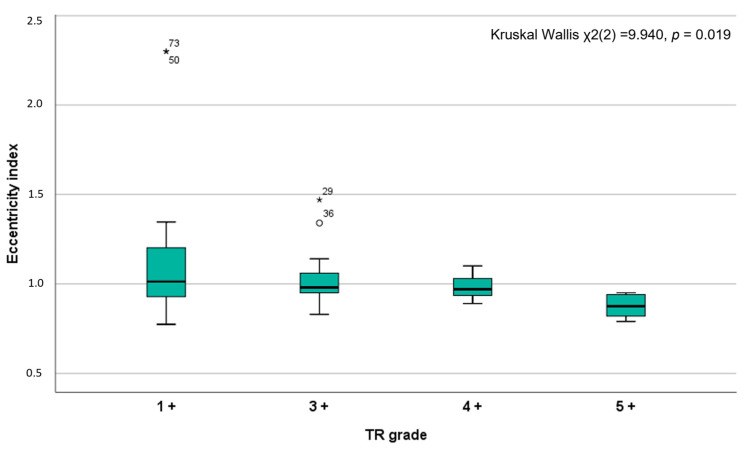
Boxplot showing the distribution of eccentricity values among the expanded tricuspid regurgitation severity grades. TR: tricuspid regurgitation.

Pearson’s correlation showed a significant relationship between all annulus measurements and the grade of TR, except for the angles γ and β (Table S2). Similarly, a stepwise increase in the CT scan values was observed across the expanded TR grades by echocardiography, except for angles and eccentricity (Table S3). Specifically, the eccentricity index progressively reduced across the TR severity grades (Figure 2).

**Figure 3:**
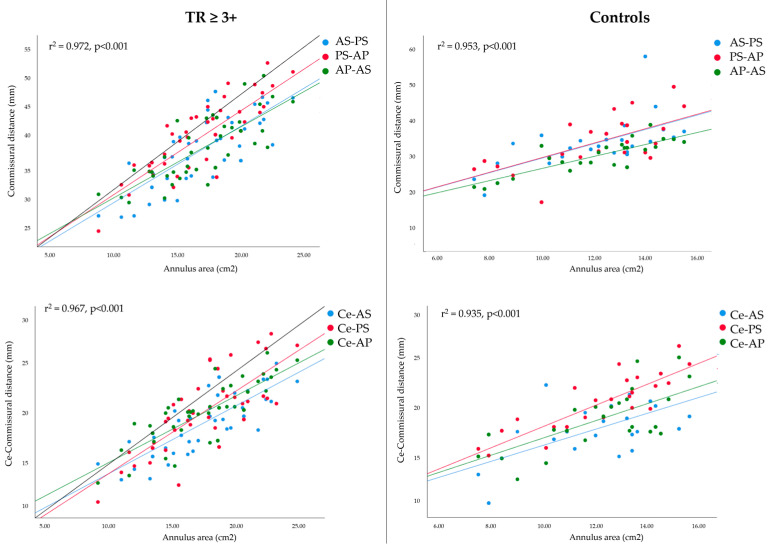
Multiple regression lines showing the relationship between CT dimensions (distance between commissures and segments between centroid and commissure) in patients with TR ≥ 3+ (left panel) and controls (right panel). AS: antero-septal commissure; AP: antero-posterior commissure; Ce: centroid; PS: postero-septal commissure; TV: tricuspid valve.

Multiple regression revealed that novel CT variables focusing on commissures predicted three-dimensional annulus area, both in patients with TR ≥ 3+ and in controls. The analysis showed a strong correlation between commissural distance and TV annulus area (r^2^ = 0.936) and between centroid-commissural distance and TV annulus area (r^2^ = 0.925). All variables added statistical significance to the prediction (Table 4). A significant correlation was also maintained within the two groups (Figure 3).

## 4. Discussion

The present study underlines the role of cardiac CT in providing a comprehensive anatomical description and illustration of the TV geometry. Notably, we observed that: (1) dedicated CT contrast protocols tailored to ensure optimal right heart opacification allow the easy identification of commissures based on their specific anatomic landmarks; (2) novel CT variables describing the TV morphology correlate significantly with conventional annulus measurements in both patients with severe TR and control subjects; (3) severe functional TR is associated with geometrical remodeling of the TV geometry; and (4) a significant relationship exists between CT measurements and the grade of TR assessed with echocardiography.

### 4.1. CT Assessment of TV Components

Due to high spatial resolution, cardiac CT provides three-dimensional data of the TV apparatus and allows a non-invasive reconstruction of the geometrical changes occurring in patients with severe TR and candidates for TTVI. Specific protocols for CT acquisition, dedicated to the right heart chambers, are needed for optimizing the image quality for analysis [8,15,22,23]. In addition, several CT parameters have been tested that specifically support different transcatheter therapies [11,12,13,14,15]. Our contrast media protocol is specifically tailored to our institutional scanners and adapted from previously published methods [15,23]. Furthermore, in our CT analysis, we introduced the study of novel TV components, which are able to provide further information about the morphology of the TV apparatus, as previously tested in a cohort of patients with severe TR [14]. In the present study, we demonstrated that these novel CT parameters focusing on commissures predict three-dimensional annulus area both in patients with TR ≥ 3+ and normal subjects, adding value to these anatomical findings.

The focal point of our analysis is on the identification of commissures based on their anatomical landmarks. Currently available software dedicated to the TV reconstruction provides automated identification of commissures, but this must be corrected according to specific anatomical findings. In particular, the commissures are not points that open directly into the annulus, but they look like small scallops or commissural leaflets. In addition, they are supported by fan-shaped chords and relative papillary muscles [24,25,26,27]. Pathological and surgical studies have differently described anatomical variants in the number and location of leaflets, but separating leaflets from scallops (and thus true commissures from clefts or indentations) is tricky by imaging techniques, especially with echocardiography. Moreover, these findings may have important clinical implications in TTVI planning and outcomes [26,27,28,29,30,31]. For this reason, knowledge of anatomical landmarks and application of this information in CT protocols is critical to the management of candidates for TTVI. In our study population, including patients with significant TR and controls, we identified a typical three-leaflet configuration by CT scan analysis in all cases. Our contrast protocols allowed adequate contrast of the right heart to detect the major AS, PS, and AP commissures. Based on the above-mentioned anatomical landmarks, no true supernumerary leaflets were detected, although multi-scalloped anterior and posterior leaflets (thus indentations on the anterior and posterior leaflets) were identified in 41.8% of cases. Once the commissures are identified, the other novel components of TV are simply measured in the short axis (transverse) view, namely distance between commissures, segments between geometric TV centroid and commissures, and angles formed by the centroid and two adjacent commissures [14].

### 4.2. TV Annulus Remodelling

The typical morphology of the TV annulus is characterized by a nonplanar saddle-shaped configuration with two high points in the antero-septal and postero-lateral region and two low points in the antero-lateral and postero-septal area [32,33]. In patients with functional TR, the three-dimensional configuration of the TV annulus changes, becoming more circular, typically flatter, and less variable between systole and diastole [11,14,33,34]. In addition, in patients with significant TR, the area of the TV annulus is typically larger than in normal subjects, as reported in a three-dimensional echocardiographic study of Ton-Nu et al. and a CT study of van Rosendael PJ et al. (1724 + 475 vs. 983 + 218 mm^2^, *p* = 0.001 and 1539.7 ± 260.2 vs. 1228.4 ± 243.5 mm^2^, *p* < 0.001, respectively) [11,33]. The present evaluation supports these previous studies. By using CT, we found that patients with TR ≥ 3+ (91% atrial functional) had larger tricuspid annulus areas and perimeters compared to patients with traces of mild TR. Similarly, septal-lateral and antero-posterior annular dimensions were larger in patients with significant TR than in controls. It is noteworthy that both diameters were similar in the TR ≥ 3+ group, resulting in an eccentricity index of 0.99 ± 0.12, whereas in the control group the septal-lateral dimension was the smallest, with an eccentricity index >1. These results are suggestive of an almost perfectly circular shape of the TV annulus in patients with significant functional TR and an oval shape in patients without significant TR.

In the present study, the geometrical remodeling of the TV apparatus in patients with functional TR compared with control is endorsed by significant changes in novel CT parameters, focusing on commissures. To the best of our knowledge, this is the first study comparing these novel CT parameters in patients with and without significant TR. In particular, all commissural distances and Ce-commissural segments were larger in patients with severe TR. Of note, the greatest difference was in the PS-AP and AP-AS distance, confirming that in the case of severe functional TR, the remodeling of the tricuspid valve is asymmetric and more pronounced along the free wall, moving the AP commissure away from the center of the TV [11,33].

### 4.3. TR Grade Correlation

Even if the functional TR grade is strongly influenced by the right ventricle preload, afterload, and right ventricle function, the association between TV geometry and TR grade has been extensively studied [11,33,35,36,37,38,39]. Ton-Nu et al., using echocardiography, demonstrated that TV annulus area and circularity were significantly associated with TR grade, while van Rosendael, P.J., et al., using CT, showed that only the antero-posterior tricuspid annulus diameter was independently correlated with TR ≥ 3+ [11,33]. The present evaluation demonstrates that all CT measurements significantly correlated with TR, showing a stepwise increase in the CT values across the expanded TR grades by echocardiography, except for eccentricity and angles. Notably, the eccentricity index decreased across the TR grades, indicating an anticipated geometric change from an oval to a circular configuration of the TV annulus when significant TR occurs. Interestingly, we did not find any correlation between the angles and the TR grade, nor significant differences in Ce-commissure angles between patients with significant TR and controls. We can therefore assume that the position of the commissures in relation to the TV centroid is stable and not influenced by either the remodeling of the annulus or the severity of TR.

### 4.4. Clinical Implications

The present study offers a different perspective for studying TV geometry in patients with functional TR and candidates for TTVI, starting from identifying commissures according to their anatomical landmarks. These novel CT variables correlate significantly with parameters currently used for tricuspid annular analysis, both in patients with significant functional (atrial) TR and in normal subjects. Moreover, these findings correlate significantly with the grade of TR assessed by echocardiography. Consequently, the extent of TV remodeling using standard and novel parameters may help in grading TR, especially when RV function, preload, and afterload, influence the occurrence and severity of functional TR. Finally, they can be used in the perspective of achieving a more complete analysis of the TV structure, thus supporting the growth and development of new transcatheter therapies.

### 4.5. Study Limitations

Even if this is the first analysis testing novel CT measurements focusing on commissures in patients with and without severe TR, the main limitation is the single-site data collection and the small sample size. The control group comprised patients referred for chest pain and undergoing triple rule-out CT protocol for acute coronary artery disease, aortic dissection, or pulmonary embolism. Patients with moderate or greater TR were not included in the control group, and other exclusion criteria were also applied in order to select a population of apparently healthy subjects. CT scan protocol differed between the two populations, even if no patients were excluded due to suboptimal CT images for evaluating TV geometry. For all these reasons, this study must be considered a preliminary and hypothesis-generating study. Our future research goals are to expand the sample size of our study population and analyze CT findings in relation to TTVI outcomes, as well as to test these results with regard to echocardiographic parameters for defining TR grade and right chamber remodeling. In addition, larger-sized CT studies could better clarify the TV geometric differences across the entire spectrum of TR etiologies [21].

## 5. Conclusions

Novel CT variables focusing on commissures increase the anatomical understanding of the TV apparatus and the TV geometrical changes in patients with severe functional TR compared to normal subjects. In particular, TR patients had larger TV annular dimensions and distance between commissures and centroid-commissural segments, without variations in the centroid-commissural angles. All measurements except for angles were able to discriminate TR severity by TEE. Therefore, these findings have the potential to support emerging transcatheter procedures by providing invaluable anatomical and geometrical information.

## Figures and Tables

**Table 1:** Overview of the CT scan parameters analyzed offline.

Parameter	Method	Description
TV annulus area	Semi-automated	TV annulus area (cm^2^)
TV annulus perimeter	Semi-automated	TV annulus perimeter (mm)
Septal-lateral diameter	Manual	Septal-lateral dimension of the TV annulus (mm), measured as the maximal distance in septal to lateral direction and coinciding with the annulus measurement in the four-chamber view
Antero-posterior diameter	Manual	Antero-posterior dimension of the TV annulus (mm), obtained orthogonally to the septal-lateral diameter and corresponding with the measurement in the two-chamber view
Eccentricity index	Manual	Ratio between the antero-posterior/septal-lateral diameter, it expresses the shape of the TV annulus
AS-PS	Automated	Distance between the antero-septal and postero-septal commissure (mm)
PS-AP	Automated	Distance between the postero-septal and antero-posterior commissure (mm)
AP-AS	Automated	Distance between the antero-posterior and antero-septal commissure (mm)
Ce-AS	Manual	Segment between the centroid and the antero-septal commissure (mm)
Ce-PS	Manual	Segment between the centroid and the postero-septal commissure (mm)
Ce-AP	Manual	Segment between the centroid and the antero-posterior commissure (mm)
α	Manual	Angle between the segments Ce-AS and Ce-PS (°)
β	Manual	Angle between the segments Ce-PS and Ce-AP (°)
γ	Manual	Angle between the segments Ce-AP and Ce-AS (°)

AP: antero-posterior; AS: antero-septal; Ce: centroid; PS: postero-septal; TV: tricuspid valve.

**Table 2:** Baseline clinical characteristics.

	TR ≥ 3+ (n = 43)	Control (n = 43)	*p*
Age, years *±* SD	78.07 ± 7.69	71.65 ± 13.67	0.009
Male, n (%)	12 (28)	24 (56)	0.009
BMI, kg/m^2^ ± SD	24.4 ± 3.89	23.22 ± 4.12	0.176
Atrial fibrillation, n (%)	39 (91)	7 (16)	<0.001
Hypertension, n (%)	25 (58)	22 (51)	0.665
Diabetes, n (%)	10 (23)	7 (16)	0.588
Dyslipidemia, n (%)	22 (51)	18 (42)	0.51
LVEF, % ± SD	51.28 ± 3.89	53.87 ± 8.53	0.074
Permanent PM/ICD/CRT	18 (41)	0	<0.001

BMI: body mass index; CRT: cardiac resynchronization therapy; ICD: implantable cardioverter-defibrillator; LVEF: left ventricular ejection fraction; PM: pacemaker; SD: standard deviation; TR: tricuspid regurgitation.

**Table 3:** Tricuspid valve measurements and differences between groups.

	TR ≥ 3+ (n = 43)	Control (n = 43)	Difference (95% CI)	*p*
Annulus area (cm^2^ ± SD)	16.84 ± 3.55	12.09 ± 2.37	4.75 (3.45 to 6.05)	<0.001
Annulus perimeter (mm ± SD)	147.77 ± 17.15	129.47 ± 13.24	18.29 (11.72 to 24.86)	<0.001
SL diameter (mm ± SD)	46.80 ± 6.44	36.80 ± 5.21	10 (7.49 to 12.51)	<0.001
AP diameter (mm ± SD)	45.67 ± 5.05	39.39 ± 4.97	6.28 (4.13 to 8.43)	<0.001
Eccentricity (index ± SD)	0.99 ± 0.12	1.11 ± 0.31	−0.12 (−0.22 to −0.02)	0.022
AS-PS (mm ± SD)	38.64 ± 5.67	35.06 ± 6.83	3.58 (0.89 to 6.27)	0.010
PS-AP (mm ± SD)	40.71 ± 5.74	35.23 ± 6.59	5.49 (2.83 to 8.14)	<0.001
AP-AS (mm ± SD)	38.80 ± 5.83	31.77 ± 4.36	7.03 (4.50 to 9.06)	<0.001
Ce-AS (mm ± SD)	21.98 ± 2.90	18.32 ± 3.12	3.66 (2.37 to 4.96)	<0.001
Ce-PS (mm ± SD)	23.02 ± 3.77	20.88 ± 3.04	2.14 (0.67 to 3.61)	0.005
Ce-AP (mm ± SD)	22.94 ± 2.84	19.22 ± 3.03	3.73 (2.46 to 4.98)	<0.001
α (° ± SD)	117.91 ± 9.43	121.81 ± 9.49	−3.91 (−7.96 to 0.15)	0.09
β (° ± SD)	123.49 ± 8.05	124.05 ± 13.16	−0.56 (−5.31 to 4.19)	0.815
γ (° ± SD)	118.14 ± 8.69	114.14 ± 8.33	4.00 (0.35 to 7.66)	0.032

AP: antero-posterior; AS: antero-septal; Ce: centroid; CI: confidence interval; PS: postero-septal; SD: standard deviation; SL: septo-lateral.

**Table 4:** Linear multiple regression analysis of CT variables associated with three-dimensional annulus area: estimated model coefficients and statistical significance of the independent variables.

	Coefficient	95% CI	*p*
AS-PS	0.146	0.105 to 0.188	<0.001
PS-AP	0.215	0.174 to 0.256	<0.001
AP-AS	0.349	0.299 to 0.399	<0.001
Ce-AS	0.497	0.415 to 0.578	<0.001
Ce-PS	0.273	0.181 to 0.366	<0.001
Ce-AP	0.479	0.385 to 0.573	<0.001

AS: antero-septal commissure; AP: antero-posterior commissure; CI: conficence interval; Ce: centroid; PS: postero-septal commissure.

## Data Availability

The data presented in this study are available on request from the corresponding author.

## Supplementary Materials

The following supporting information can be downloaded at: https://www.mdpi.com/article/10.3390/jcm12051712/s1, Table S1: Tricuspid valve measurements and differences between patients with pure functional TR and CIED-induced TR; Table S2: Pearson correlation between CT variables and TR grade assessed with echocardiography; Table S3: Kruskal-Wallis analysis testing the CT values distribution across the expanded TR grades by echocardiography.
