# Supplementary material for: Tricuspid Valve Geometrical Changes in Patients with Functional Tricuspid Regurgitation: Insights from a CT Scan Analysis Focusing on Commissures
Source: J Clin Med. 2023 Feb 21;12(5):1712. doi: 10.3390/jcm12051712 (PMC10003433; doi:10.3390/jcm12051712)
Supplement: Supplementary file 1 [file jcm-12-01712-s001.zip › jcm-2168313-supplementary.pdf]

Supplementary Materials:

Table S1. Tricuspid valve measurements and differences between patients with pure functional TR and CIED-induced TR.

	Funtional TR (n=25)	CIED-TR (n=18)	Difference (95% CI)	p
Annulus area (cm2 ± SD)	16.35 ± 3.12	17.51 ± 4.09	-1.15 (-3.37 to 1.07)	0.301
Annulus perimeter (mm ± SD)	144.87 ± 14.66	151.79 ± 19.85	-6.92 (-17.53 to 3.70)	0.196
SL diameter (mm ± SD)	46.02 ± 6.40	47.88 ± 6.50	-1.86 (-5.89 to 2.16)	0.355
AP diameter (mm ± SD)	45.42 ± 4.58	46.03 ± 5.76	-0.61 (-3.79 to 2.58)	0.702
Eccentricity (index ± SD)	1.00 ± 0.14	0.97 ± 0.09	0.03 (-0.04 to 0.11)	0.380
AS-PS (mm ± SD)	37.88 ± 5.36	39.70 ± 6.07	-1.83 (-5.37 to 1.70)	0.302
PS-AP (mm ± SD)	39.61 ± 5.51	42.25 ± 5.86	-2.64 (-6.17 to 0.89)	0.139
AP-AS (mm ± SD)	38.34 ± 4.58	39.44 ± 5.79	-1.10 (-4.30 to 2.24)	0.489
Ce-AS (mm ± SD)	21.64 ± 2.55	22.46 ± 3.35	-0.82 (-2.64 to 1.10)	0.368
Ce-PS (mm ± SD)	22.43 ± 3.68	23.84 ± 3.87	-1.41 (-3.75 to 0.97)	0.232
Ce-AP (mm ± SD)	22.41 ± 2.45	23.69 ± 3.23	-1.28 (-3.03 to 0.47)	0.147
α (° ± SD)	118.56 ± 8.69	117.00 ± 10.57	1.56 (-4.38 to 7.50)	0.599
β (° ± SD)	121.91 ± 8.59	125.67 ± 7.85	-3.75 (-8.92 to 1.43)	0.152
γ (° ± SD)	118.72 ± 9.03	117.33 ± 8.38	1.39 (-4.08 to 6.86)	0.611

AP: antero-posterior; AS: antero-septal; Ce: centroid; CI: confidence interval; CIED: cardiac implantable electronic device; PS: postero-septal; SD: standard deviation; SL: septo-lateral; TR: tricuspid regurgitation

Table S2. Pearson correlation between CT variables and TR grade assessed with echocardiography

	Coefficient r	p
Annulus area	0.580	<0.001
Annulus perimeter	0.468	<0.001
Septal-lateral dimension	0.646	<0.001
Antero-posterior dimension	0.462	<0.001
Eccentricity	-0.277	0.005
AS-PS	0.256	<0.001
PS-AP	0.389	<0.001
AP-AS	0.557	<0.001
Ce-AS	0.470	<0.001
Ce-PS	0.286	0.04
Ce-AP	0.526	<0.001
α	-0.166	0.063
β	-0.46	0.338
γ	0.207	0.028

AP: antero-posterior; AS: antero-septal; Ce: centroid; CI: confidence interval; PS: postero-septal; SD: standard deviation; SL: septo-lateral

Table S3. Kruskal-Wallis analysis testing the CT values distribution across the expanded TR grades by echocardiography

	H	p
Annulus area	33,007	<0.001
Annulus perimeter	21,921	<0.001
Septal-lateral dimension	40,204	<0.001
Antero-posterior dimension	25,322	<0.001
Eccentricity	9,940	0.019
AS-PS	12,107	0.007
PS-AP	17,038	0.001
AP-AS	31,967	<0.001
Ce-AS	23,402	<0.001
Ce-PS	11,034	0.012
Ce-AP	25,254	<0.001
α	3,901	0.272
β	1,603	0.659
γ	6,900	0.075

AP: antero-posterior; AS: antero-septal; Ce: centroid; CI: confidence interval; PS: postero-septal; SD: standard deviation; SL: septo-lateral
